# “Polygenic Genetic Risk Factors in Alzheimer’s Disease: A Case‐Control Study in Peruvian Population”

**DOI:** 10.1002/alz.092361

**Published:** 2025-01-03

**Authors:** Jonathan Adrian Zegarra‐Valdivia, Harold Alessandro Arana‐Nombera, Leandro Aron Perez‐Fernandez, Milagros del Rocio Casimiro‐Arana, Reyna Elizabeth Alamo‐Medina, Viviana Nayelli Gallegos‐Manayay, María del Rosario Oliva‐Piscoya, Nobuko Vásquez‐Zuñe, Eduardo Sebastián Abanto‐Saldaña, Maria Celinda Cruz Ordinola, Carmen Paredes Manrique, Miguel E. Renteria, Sheila Castro‐Suarez, Neuroscience and Mental Health Group

**Affiliations:** ^1^ Universidad Señor de Sipán, Chiclayo Peru; ^2^ Universidad San Martín de Porres, Chiclayo Peru; ^3^ Universidad Tecnológica del Perú, Arequipa Peru; ^4^ Global Brain Health Institute, San Francisco, CA USA; ^5^ QIMR Berghofer Medical Research Institute, Brisbane, QLD Australia; ^6^ Instituto Nacional de Ciencias Neurologicas, Lima Peru; ^7^ Global Brain Health Institute (GBHI), University of California San Francisco, San Francisco, CA USA

## Abstract

**Background:**

Alzheimer’s Disease (AD) is the most prevalent form of dementia globally. While some familial cases are observed, sporadic AD cases are more common and reflect a high level of complexity, with individual risk determined by the interaction of polygenic and environmental factors.

**Objective:**

To characterize polygenic genetic risk factors in individuals with cognitive impairment and Alzheimer’s Disease across four regions of Peru.

**Method:**

This will be a prospective observational case‐control study. We will identify the neurocognitive profile of participants and collect saliva samples for subsequent genetic profiling. Participants will be divided into three main groups: those with cognitive impairment, those diagnosed with AD, and control subjects over 50 years old without dementia.

**Results:**

We expect to validate the effect of risk variants previously identified in the European population within the Peruvian population. Additionally, the aim is to identify new genetic risk factors specific to the Peruvian population. Collectively, the findings from this study could lay the groundwork for implementing a polygenic risk score model, which would estimate an individual’s genetic risk of developing Alzheimer’s Disease.

**Conclusion:**

This study aims to significantly contribute to the understanding of Alzheimer’s Disease’s genetic underpinnings in the Peruvian population. By exploring both known and novel genetic risk factors, we hope to enhance early detection strategies and personalized risk assessments for AD, ultimately guiding more targeted interventions and preventative measures.

